# Obturators and Artificial Vela

**Published:** 1885-06

**Authors:** N. W. Kingsley


### OBTURATORS AND ARTIFICIAL VELA.

BY N. W. KINGSLEY, D. D. S.

An article in the May number of the INDEPENDENT PRACTITIONER, on "Obturators," written by a young man who has just graduated at the New York College of Dentistry, contains errors which, unless corrected immediately, may obtain some credence for want of denial.

On page 244 he says: "Dr. Kingsley, at the meeting of the First District Dental Society for February, 1885, remarked that no one could construct an obturator for a patient with a congenital cleft, in which articulation was noticeably improved during the first month's wear."

I said nothing of the kind. What I did say was that "no one could construct an apparatus for a congenital cleft which would restore perfect articulation *immediately*." (Vide report of that meeting in *Cosmos* for April, page 227.)

The aim of Dr. Weber's article is to make it appear that an obturator is applicable in *all* cases of congenital cleft, and that with an obturator a patient can *always* acquire articulation as quickly as with any other kind of an appliance. Such an assumption as this, by a young man who has never seen artificial vela put to a test in comparison with obturators, is simply amazing. My own experience has extended over a quarter of a century, with all kinds of appliances, and for hundreds of patients, and I reiterate the quotation from my writings, made by Dr. Weber at the head of page 244, with the added force of additional years of experience.

Obturators can certainly be used in some cases, with reasonable expectation that the patient will derive the desired benefit as soon as with any other instrument; but such cases are only those where, from habit or otherwise, there is already great activity of the pharyngeal muscles in the effort to articulate. In many instances, these muscles in that effort seem quite dormant, and for these latter cases artificial *vela* are at present the only known remedy.

Dr. Weber would intimate that all of Suersen's patients had acquired articulation by that means, and yet a little over a year ago there came into my hands a young lady who had worn one of Suersen's obturators for several years (made by himself), whose articulation had not been benefited. I made for her an artificial velum, in January, 1884. I have seen her within two months, and her speech is now perfect. She is now in condition to illustrate the second paragraph quoted by Dr. Weber on page 244, viz.: "That a soft, elastic, artificial velum is much better adapted to the acquirement of articulation than one of an unyielding, non-elastic substance, *but when acquired* an obturator may be substituted." The philosophy of this statement is very simple. All experience shows that a patient whose speech has been perfect, and who has lost it by an acquired lesion of the palate, will have that speech instantly restored by almost any very simple form of an obturator. The former faculty of speech returns immediately after the lesion is closed. Congenitally cleft patients, having acquired the faculty of distinct articulation with an artificial velum, can continue to exercise that faculty with a properly adjusted obturator.

